# Draft genome and de novo transcriptome assembly of the Antarctic marine flatworm *Obrimoposthia wandeli*

**DOI:** 10.1186/s12863-025-01390-7

**Published:** 2025-12-02

**Authors:** Seung Chul Shin, Sanghee Kim

**Affiliations:** Division of Life Sciences, Korea Polar Research Institute (KOPRI), Incheon, 21990 Republic of Korea

**Keywords:** *Obrimoposthia wandeli*, Antarctica, Draft genome assembly

## Abstract

**Objectives:**

*Obrimoposthia wandeli* is the most abundant marine planarian in the intertidal zone of the maritime Antarctic region and is presumed to have adapted to the extreme conditions of the Southern Ocean. However, genomic studies on marine flatworms remain extremely limited, with only one marine planarian genome currently available in public databases. We present the first draft genome and de novo transcriptome assembly of *O. wandeli* from Antarctica, providing a valuable genomic resource for the study of flatworm biology and environmental adaptation under harsh environments.

**Data description:**

We sequenced the genome and transcriptome of *O. wandeli* collected near King Sejong Station using Oxford Nanopore long-read, Illumina paired-end, and RNA sequencing. The draft genome assembly spans 1.35 Gb across 6,912 contigs, with an N50 of 343,088 bp and an estimated 48,310 predicted genes. The assembly showed Benchmarking Universal Single-Copy Orthologs (BUSCO) completeness scores of 80.2% (genome) and 80.8% (proteins). The de novo transcriptome assembly identified 26,169 non-redundant transcripts, with a BUSCO completeness of 93.1%. These genomic and transcriptomic datasets represent the first Antarctic marine planarian reference and provide a valuable resource for studying marine flatworm genomics.

## Objective

*Obrimoposthia wandeli* is the most abundant planarian flatworm found along the maritime coastal area near King Sejong Station in Antarctica [1, 2]. However, minimal molecular data are available for *O. wandeli*; to date, only a single ribosomal sequence (MH108586.1) and mitochondrial genome (NC_050050.1) have been reported [3]. Planarians belonging to Platyhelminthes, Tricladida, are renowned for their regenerative abilities [4]. Although reference genome sequences for nine planarian species have been deposited in NCBI, most are freshwater (*n* = 5) or terrestrial (*n* = 3) species. Among the over 1,700 known tricladid species, only approximately 80 are marine, and only one marine species, *Sluysia triapertura*, has a publicly available reference genome. We sequenced the genome and transcriptome of *O. wandeli* using a combination of Oxford Nanopore long-read, Illumina paired-end, and RNA sequencing. Here, we present a draft genome assembly, gene annotation, and transcriptome assembly of *O. wandeli*, providing a valuable resource for studying marine flatworm genomics and serving as a representative reference for comparative analyses of marine species.

## Data description

*O. wandeli* were individually collected from intertidal zones areas near King Sejong Station, King George Island, Antarctica (62°12′S, 58°47′W) using sterile forceps (Table 1, Data file 1 [5]). Live flatworms were transferred to the laboratory and maintained in filtered autoclaved seawater, which was replaced daily. Before DNA and RNA extraction, the specimens were starved for one week to minimize contamination from ingested materials. To effectively removed excess mucus and minimized the risk of co-extracting environmental materials adhering to the specimen surface, 5 N NaCl and GTC buffer (4 M guanidinium thiocyanate, 25 mM sodium citrate, 0.5% v/v N-lauroylsarcosine, and 7% v/v β-mercaptoethanol) were applied. A pooled sample consisting of 10 specimens was used for genomic DNA and RNA preparation. Genomic DNA was extracted using DNeasy Tissue Kits (Qiagen, USA), while total RNA was isolated from whole-body tissue homogenized in liquid nitrogen using RNeasy Mini Kits (Qiagen). Sequencing was performed using Illumina HiSeq and Oxford Nanopore PromethION platforms (Table 1, Data file 2 [5]). Illumina sequencing yielded 103.2 Gb of genomic reads and 8.9 Gb of RNA-seq reads, while Nanopore sequencing generated 69.2 Gb of long reads (Table 1, Data files 3–5 [6]).

**Table 1:** Overview of data files/data sets

Label	Name of data file/data set	File types (file extension)	Data repository and identifier (DOI or accession number)
*Data file 1*	Table 1, *Data File 1. Obrimoposthia wandeli* on a beach near the King Sejong Station in Antarctica	*Microsoft Word (.docx)*	Figshare (10.6084/m9.figshare.29499194) [5]
*Data file 2*	Table 1, *Data File 2.* Sequencing read statistics	*Microsoft Word (.docx)*	Figshare (10.6084/m9.figshare.29499194) [5]
*Data file 3*	Illumina reads of *O. wandeli* genomic DNA	*Fastq file (.fastq)*	NCBI SRA (https://identifiers.org/insdc.sra:SRP599601) [6]
*Data file 4*	RNA-Seq of *O. wandeli* (RNA)	*Fastq file (.fastq)*	NCBI SRA (https://identifiers.org/insdc.sra:SRP599601) [6]
*Data file 5*	ONT sequence reads of *O. wandeli* genomic DNA	*Fastq file (.fastq)*	NCBI SRA (https://identifiers.org/insdc.sra:SRP599601) [6]
*Data file 6*	Table 1, *Data File 6.* Genome size prediction.	*Microsoft Word (.docx)*	Figshare [5]: (10.6084/m9.figshare.29499194)
*Data file 7*	*O. wandeli* Genomic contigs	*Fasta file (.fasta)*	Figshare (10.6084/m9.figshare.29499194) [5] and ENA (https://www.ebi.ac.uk/ena/browser/view/GCA_977020255) [7]
*Data file 8*	Table 1, *Data File 8.* Genome assembly statistics	*Microsoft Word (.docx)*	Figshare (10.6084/m9.figshare.29499194) [5]
*Data file 9*	Table 1, *Data File 9.* BUSCO completeness and repeat composition	*Microsoft Word (.docx)*	Figshare (10.6084/m9.figshare.29499194) [5]
*Data file 10*	Table 1, Data File 10. Mitochondrial genome sequence of *O. wandeli* obtained in this study	*Fasta file (.fasta)*	Figshare (10.6084/m9.figshare.29499194) [5] and ENA (https://www.ebi.ac.uk/ena/browser/view/GCA_977020185) [8]
*Data file 11*	Table 1, *Data File 11.* Dot plot between the mitochondrial genome sequences of *O. wandeli*	*Microsoft Word (.docx)*	Figshare (10.6084/m9.figshare.29499194) [5]
*Data set 12*	Table 1, *Data File 12*. Repeat Annotation statistics	*Microsoft Word (.docx)*	Figshare (10.6084/m9.figshare.29499194) [5]
*Data file 13*	Table 1, *Data File 13*. Gene Annotation statistics	*Microsoft Word (.docx)*	Figshare (10.6084/m9.figshare.29499194) [5]
*Data file 14*	protein sequences annotated from BRAKER3	*Fasta file (.fasta)*	Figshare (10.6084/m9.figshare.29499194) [5]
*Data file 15*	Coding sequences annotated from BRAKER3	*Fasta file (.fasta)*	Figshare (10.6084/m9.figshare.29499194) [5]
*Data file 16*	Annotation of gene (EggNOG and BLASTp)	*Excel file (.xlsx)*	Figshare (10.6084/m9.figshare.29499194) [5]
*Data file 17*	Table 1, *Data File 17.* Transcriptome assembly statistics	*Microsoft Word (.docx)*	Figshare (10.6084/m9.figshare.29499194) [5]
*Data file 18*	Transcript sequences of *De novo* transcriptome	*Fasta file (.fasta)*	Figshare (10.6084/m9.figshare.29499194) [5]
*Data file 19*	Protein sequences of *De novo* transcriptome (protein)	*Fasta file (.fasta)*	Figshare (10.6084/m9.figshare.29499194) [5]
*Data file 20*	Annotation of Transcriptome (EggNOG and BLASTp)	*Excel file (.xlsx)*	Figshare (10.6084/m9.figshare.29499194) [5]

Raw read quality was assessed using FastQC (v0.11.8 [9]). Genome characteristics were estimated by k-mer analysis (k = 29) using Jellyfish (v2.3.0 [10]) and GenomeScope (v1.0 [11]), indicating a genome size of approximately 1.12 Gb, heterozygosity rate of 0.25%, and repeat content of 51.5% (Table 1, Data file 6 [5]). Genome assembly was performed using SMARTdenovo (RRID: SCR_017622 [12]) with Nanopore reads, and polished with NextPolish (v1.3.1 [13]) using high-quality Illumina data. The final genome assembly comprised 6,912 contigs totaling 1.35 Gb, with an N50 of 343,088 bp and a maximum contig length of 4.677 Mb (Table 1, Data files 7–8 [5, 7]). Benchmarking Universal Single-Copy Ortholog (BUSCO) (v5.1.3 [14]) analysis of the *eukaryota_*odb10 dataset indicated 80.2% completeness (Table 1, Data File 9a [5]). The resulting genome assembly and BUSCO completeness of *O. wandeli* were compared with those of other available planarian genomes (Table 1, Data files 8–9a [5]), including one marine species (*Sluysia triapertura*, GCA_046118255.1) and two freshwater species (*Schmidtea mediterranea*, GCA_045838265.1; *Schmidtea nova*, GCA_044892505.1) [15, 16]. The *O. wandeli* assembly was more fragmented than the chromosome-level assemblies of *S. mediterranea* and *S. nova*, both of which were scaffolded using Hi-C data (four and three chromosomes, respectively). However, the *S. triapertura* assembly generated solely from short-read data was substantially more fragmented than that of *O. wandeli* (Table 1, Data File 8–9a [5]). The mitochondrial genome sequence was also obtained to confirm the species identification as *O. wandeli*. For mitochondrial genome assembly, Illumina reads were mapped to the reference mitochondrial genome of *O. wandeli* (NC_050050.1) using Bowtie2 (v2.4.5) [17] and reassembled using Unicycler (v0.4.8) [18]. The resulting circular contig was 15,060 bp long (Table 1, Data File 10 [5, 8]) and showed 99.59% nucleotide identity with the reference mitochondrial genome (NC_050050.1), based on BLAST2seq analysis [19]. A dot plot generated using SyMAP5 (v5.0) [20] further confirmed that the two mitochondrial genome sequences were nearly identical and collinear (Table 1, Data File 11 [5]).

Repeat elements were annotated using RepeatMasker (v4.0.7 [21]) with a custom repeat library generated by RepeatModeler (v1.0.11 [22]). Overall, 67.9% of the genome was masked as repetitive, with retroelements (18.3%) and DNA transposons (28.99%) representing the majority. Simple repeats accounted for 0.64% of the genome, and 3,497 tRNA genes were identified using tRNAscan-SE (v2.0 [23]) (Table 1, Data file 12 [5]). The resulting repeat sequences were compared with those of *S. mediterranea* and *S. nova* in terms of total length. DNA transposons and Long Terminal Repeat (LRT) elements comprised a higher proportion of annotated repeat sequences in all three species (Table 1, Data file 9b [5]).

Gene prediction was conducted using BRAKER3 [24], incorporating RNA-seq reads and protein hints from *Schmidtea mediterranea* [4, 15, 16] and the *de novo* transcriptome of *O. wandeli*. Overall, 48,310 protein-coding genes were predicted and used for functional annotation (Table 1; Data files 13–16 [5]). BUSCO analysis in the protein mode showed 80.8% annotation completeness, which was slightly lower than that of the chromosome-level assemblies of *S. nova* (88.6%) and *S. polychroa* (91.8%) (Table 1, Data file 9c [5]). Functional annotation was performed using EggNOG-mapper (v2.1.12 [25]) against the eggNOG (v5.0 [26]) database and BLASTp (v2.0.8 [27]) against the NCBI nr database (E-value ≤ 1e − 5), with 26,454 genes annotated via EggNOG and 20,533 via BLASTp (Table 1, Data file 16 [5]).

For transcriptome assembly, low-quality reads and adapter sequences were trimmed from RNA-seq reads using Trimmomatic (v0.39 [28]) with the parameters ILLUMINACLIP2:30:10 LEADING:3 TRAILING:3 SLIDINGWINDOW:4:15 MINLEN:36, and assembled *de novo* using Trinity (v2.13.1 [29]). Transcripts with TPM < 1 were removed; the remaining sequences were clustered using CD-HIT-EST (v4.8.1 [30]) at 90% identity, retaining ≥300 bp length. Transcript abundance was quantified by mapping the reads back to the unigene set using Bowtie2 (v2.5.4 [17]), followed by quantification with RSEM (v1.3.3 [31]). Coding regions were predicted using TransDecoder (v5.7.1 [32]), identifying 26,169 non-redundant coding sequences with an average length of 1,159 bp. BUSCO analysis against the *eukaryota_odb10* dataset indicated 93.1% completeness, 4.6% missing, 2.3% fragmentation, and 17.2% duplication. Functional annotation yielded 17,217 and 21,860 annotated genes via EggNOG and BLASTp, respectively (Table 1, Data files 18–20 [5]).

## Limitations

The genomic and transcriptomic resources presented herein represent the first reference datasets for an Antarctic marine planarian, and serve as representative resources for the comparative analyses of marine species. Although the transcriptome assembly showed high completeness (93.1% complete BUSCOs), the draft genome and genes predicted by BRAKER3 exhibited lower completeness (80.2% and 80.8%, respectively), indicating room for improvement. The Oxford Nanopore sequencing data provided approximately 67× coverage, which was sufficient for a draft genome but may be inadequate for producing a more contiguous assembly. Additional long-read sequencing and/or Hi-C scaffolding would be beneficial to reduce the number of contigs and improve both assembly contiguity and gene annotation quality.

## Data Availability

The data described in this Data note are freely and openly available in NCBI under BioProjectID PRJNA1289081, in ENA under BioProjectID PRJEB102494, and in Figshare. Details and access links are provided in Table 1 and references [5–8].

## Abbreviations

BUSCO: BUSCO Benchmarking Universal Single-Copy Orthologs
TPM: TPM Transcripts Per Million
HI-C: HI-C High-throughput chromosome conformation capture
